# Quantitative analyses of the hepatic proteome of methylmercury-exposed Atlantic cod (*Gadus morhua*) suggest oxidative stress-mediated effects on cellular energy metabolism

**DOI:** 10.1186/s12864-016-2864-2

**Published:** 2016-08-05

**Authors:** Fekadu Yadetie, Silje Bjørneklett, Hilde Kristin Garberg, Eystein Oveland, Frode Berven, Anders Goksøyr, Odd André Karlsen

**Affiliations:** 1 Department of Biology, University of Bergen, PO Box 7803, N-5020 Bergen, Norway; 2 Department of Biomedicine, Proteomics Unit (PROBE) at the University of Bergen, Bergen, Norway

## Abstract

**Background:**

Methylmecury (MeHg) is a widely distributed environmental pollutant with considerable risk to both human health and wildlife. To gain better insight into the underlying mechanisms of MeHg-mediated toxicity, we have used label-free quantitative mass spectrometry to analyze the liver proteome of Atlantic cod (*Gadus morhua*) exposed *in vivo* to MeHg (0, 0.5, 2 mg/kg body weight) for 2 weeks.

**Results:**

Out of a toltal of 1143 proteins quantified, 125 proteins were differentially regulated between MeHg-treated samples and controls. Using various bioinformatics tools, we performed gene ontology, pathway and network enrichment analysis, which indicated that proteins and pathways mainly related to energy metabolism, antioxidant defense, cytoskeleton remodeling, and protein synthesis were regulated in the hepatic proteome after MeHg exposure. Comparison with previous gene expression data strengthened these results, and further supported that MeHg predominantly affects many energy metabolism pathways, presumably through its strong induction of oxidative stress. Some enzymes known to have functionally important oxidation-sensitive cysteine residues in other animals are among the differentially regulated proteins, suggesting their modulations by MeHg-induced oxidative stress. Integrated analysis of the proteomics dataset combined with previous gene expression dataset showed a more pronounced effect of MeHg on amino acid, glucose and fatty acid metabolic pathways, and suggested possible interactions of the cellular energy metabolism and antioxidant defense pathways.

**Conclusions:**

MeHg disrupts mainly redox homeostasis and energy generating metabolic pathways in cod liver. The energy pathways appear to be modulated through MeHg-induced oxidative stress, possibly mediated by oxidation sensitive enzymes.

**Electronic supplementary material:**

The online version of this article (doi:10.1186/s12864-016-2864-2) contains supplementary material, which is available to authorized users.

## Background

Mercury is released into the environment from both natural and anthropogenic sources, and occurs in different chemical forms in the environment, such as elemental mercury, inorganic mercury, and also as more toxic organic mercury compounds. The different forms of mercury can be interconverted in a global mercury cycle between the aquatic systems and the atmosphere [1]. Methylmercury (MeHg), which is an organic compound, is formed from inorganic mercury in the aquatic environment by anaerobic bacteria [2]. In aquatic environments, MeHg tends to bioconcentrate and bioaccumulate in organisms, and it has an extremely high potential for biomagnification up the food-chain [3]. MeHg can therefore affect the health of both aquatic animals and humans, as humans can be exposed to MeHg through consumption of fish and other seafood. Because of safety concerns, food safety authorities often issue guidelines for the public to avoid or limit the consumption of some fish species from contaminated environments or with high muscle mercury levels [3].

MeHg is highly toxic to animal tissues and organs, especially the brain [1, 4, 5]. Its neurotoxic properties are partly due to its easy transport throughout the body, including the blood–brain and blood-placental barriers, as a complex with cysteine [4]. The MeHg–L-cysteine complex has a similar structure as methionine and can be transported across cellular membranes by L-type amino acid transporters [6]. Its neurotoxic effects have been well documented from tragic human poisoning incidents in Japan (Minamata Bay) and Iraq (rural areas), and also from controlled laboratory experiments [5, 7, 8]. Despite the focus of many studies on the brain where most serious effects are elicited, MeHg affects other animal organs and tissues as well [1, 4, 5]. For example, in fish, effects on other tissues such as the liver and kidney have been observed [9, 10]. Further, a recent epidemiological study has reported associations between blood mercury levels and changes in liver enzymes [11]. Among the main cellular effects caused by MeHg are oxidative damage and mitochondrial dysfunction, disruption of Ca^2+^ homeostasis, apoptosis, inhibition of microtubule assembly, and inhibition of protein synthesis [5, 12–16].

The underlying molecular mechanism of MeHg toxicity seems to be related to its ability to bind thiol-groups present in biological molecules, including proteins. This may lead to disruption of enzymatic functions, and particularly inactivation of antioxidant systems, thereby promoting generation of reactive oxygen species (ROS) [12, 13]. Reactive thiol-groups of antioxidants such as glutathione and proteins in the antioxidant defense systems are known targets of MeHg-induced oxidation [13]. Indeed, MeHg has been shown to inhibit components in the two major antioxidant defense pathways, the thioredoxin and glutaredoxin enzyme systems [17–19]. Compromised antioxidant defense can potentially result in severe oxidative damage to sensitive proteins, and thus affecting related pathways.

Analyses of gene expression and proteomic responses are increasingly useful in studying the effects and toxicity mechanisms of environmental contaminants [20]. Quantitative proteomics approaches for mapping molecular targets of toxicants may lead to a better understanding of the molecular mechanisms of toxicity and discovery of new biomarkers [21]. Recent studies using proteomics have reported that MeHg causes alterations of proteins and pathways related to antioxidant defense mechanisms, energy metabolism, and the cytoskeleton in various organs and cells in both mammals [22, 23] and fish [24–27]. These studies suggest that MeHg has similar molecular mechanisms of toxicity in various tissues of different animal species. Although the liver is the most important organ in metabolism and detoxification of environmental chemicals, it has not been well investigated as a target organ of MeHg toxicity, particularly by large-scale proteomics approaches.

The Atlantic cod (*Gadus morhua)* is a commercially important species, but increasingly susceptible to marine pollution from sources such as shipping activities, urban effluent discharges, and discharges from offshore petroleum industries. Importantly, Atlantic cod is increasingly becoming a useful model organism to investigate effects of contaminants, and as a sentinel species used in environmental monitoring of marine pollution [28–34]. It is one of few fish species with a sequenced and annotated genome [35], thus opening the possibility of using omics methods in molecular toxicological studies [25, 36, 37].

The aim of this study was to investigate the molecular targets and mechanisms of toxicity of MeHg on the liver proteome of Atlantic cod. In our previous work, global hepatic gene expression analysis with microarrays was used to study the effect of MeHg on gene expression using the same samples [37]. As part of an integrated approach using both transcriptomics and proteomics, we have in this study performed label-free mass spectrometry analysis of the liver proteomes of cod exposed *in vivo* to 0, 0.5 and 2 mg/kg body weight MeHg. In the present study, 125 differentially regulated proteins were identified, and functional enrichment analysis showed that oxidative-stress responses and energy pathways were among the most affected by the MeHg treatment. These findings are in line with our previous gene expression data, and further suggest that MeHg may cause vulnerabilities of cellular energy pathways to oxidative stress.

## Results and discussion

### Fish exposure, survival and condition factor

To examine dose–response effects of MeHg on Atlantic cod, an *in vivo* exposure experiment with vehicle control and three different doses of MeHg (0.5, 2 and 8 mg/kg BW) were performed (*n* = 10/group). All fish in the highest (8 mg/kg BW) dose group, and 1 fish from each of the 0.5 and 2 mg dose groups, died by the end of the 14 days exposure time [24]. The lowest dose of MeHg (0.5 mg/kg BW) was chosen based on approximates of mercury levels in fish reported in some field studies. For example, mean mercury concentrations of up to 0.46 mg/kg wet-weight were detected in fish caught in the Northwest Atlantic Ocean, and up to 2.07 mg/kg wet-weight in fish from the Mediterranean Sea have been reported [38]. The maximum limits of Hg allowed in fish and fish products for human consumption is 0.5 or 1 mg/kg wet-weight, depending on the species [39]. Previous chemical analysis of tissue concentrations of Hg in the fish studied here, showed accumulation in the liver with average levels of 0.8 and 2.8 mg Hg/kg wet weight for the 0.5 and 2 mg/kg BW MeHg doses, respectively [24]. Higher Hg levels were detected in the muscle with 0.9 and 4.7 mg/kg wet weight for 0.5 and 2 mg/kg BW doses, respectively [24], consistent with a known tendency of MeHg to accumulate in muscle tissue [28]. No significant changes were observed in behavioural parameters such as overall activity and balance during the 2 weeks of MeHg exposure period [24]. There were small significant decreases in body weight at the end of the experiment in all groups, including the control (Additional file 1: Figure S1A). Condition factor decreased only slightly (not significant) in the treated groups (Additional file 1: Figure S1B) and there were no significant differences in liver-somatic indices between control and treated groups (Additional file 1: Figure S1C). Although the slight decrease in condition factor suggests some effects of the treatment, the observed decrease in body weight does not seem to be the main effect of MeHg treatment as it was also observed in the control group (Additional file 1: Figure S1A).

### Differentially regulated proteins

**Fig. 1:**
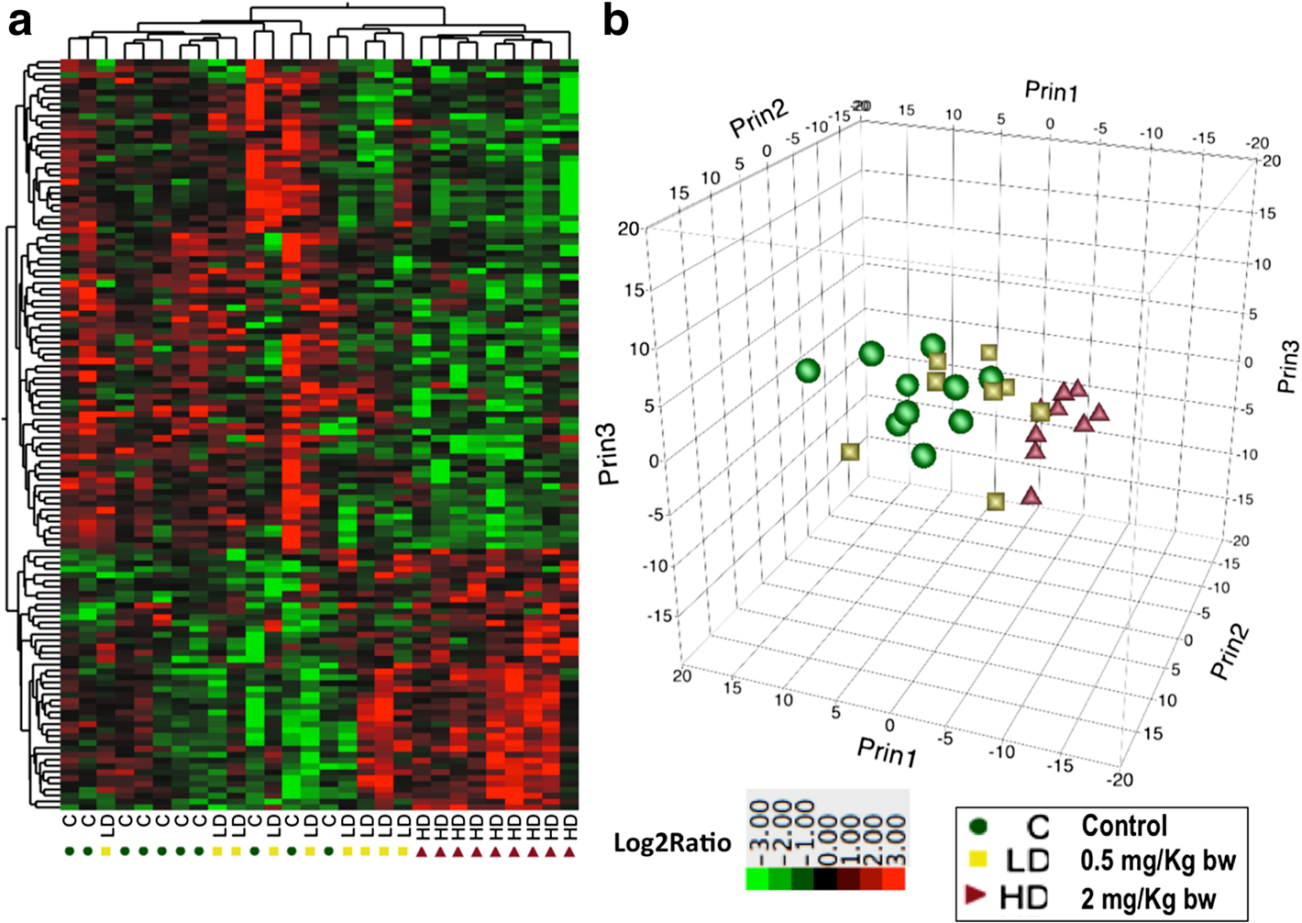
Hierarchical clustering (**a**) and principal component analysis (PCA) (**b**) of proteins differentially regulated by MeHg. Rows represent log_2_-transformed abundance ratio values of proteins, while columns represent individual cod liver samples (**a**). Three-dimensional PCA of normalised LC-MS/MS data (**b**). Individual samples are labelled according to the experimental group that they belong to; *green circles*: controls; *yellow squares*: 0.5 mg/Kg BW (LD); *red-brown* triangles: 2.0 mg/Kg BW (HD) (**a** and **b**)

Total liver protein samples from Atlantic cod exposed to 0, 0.5 or 2 mg/kg body weight MeHg were prepared and subjected to label-free mass spectrometry analyses followed by quantitative proteomics. A total of 125 proteins were differentially regulated between the high dose (2 mg/kg BW) MeHg-treated samples and controls (Additional file 2: Table S1). Peptide abundance values in all the three groups were found to have very similar profiles and resembled normal distributions (Additional file 1: Figure S2) for the 1143 proteins quantified (Additional file 3: Table S2). Among the 125 proteins differentially regulated, 44 were up-regulated and 81 down regulated (Additional file 2: Table S1). Hierarchical cluster analysis of the differentially regulated proteins shows the relative abundance ratio (fold-changes relative to control) of each protein in the individual samples (Fig. 1a). The cluster analysis shows well-clustered high dose samples separated from the other groups. However, the low dose and control samples show less within group clustering, and the two groups are less separated from each other (Fig. 1a). Of note, in the matrix data used for clustering and PCA, only 7 proteins were significantly differentially regulated in the low dose group. The three-dimensional PCA plot (Fig. 1b) shows a similar trend as observed in the hierarchical cluster analysis. The high dose samples are clearly clustered and separated apart from the controls, while samples from the low MeHg doses largely fall between high dose and control groups, more scattered among the latter group (Fig. 1b).

Among the differentially regulated proteins, many were previously shown to have redox-sensitive Cys-residues in other studies. These include the thioredoxin enzyme system proteins [18], as well as proteins that are part of the fatty acid metabolism pathways, such as mitochondrial acyl-CoA dehydrogenases [40]. In addition, chaperons, tubulin and hemoglobin subunits were among the differentially regulated proteins between MeHg-treated and control groups (Additional file 2: Table S1), which is also in line with previous two-dimensional gel electrophoresis analyses of the thiol-proteome obtained from the same liver samples [25].

**Fig. 2:**
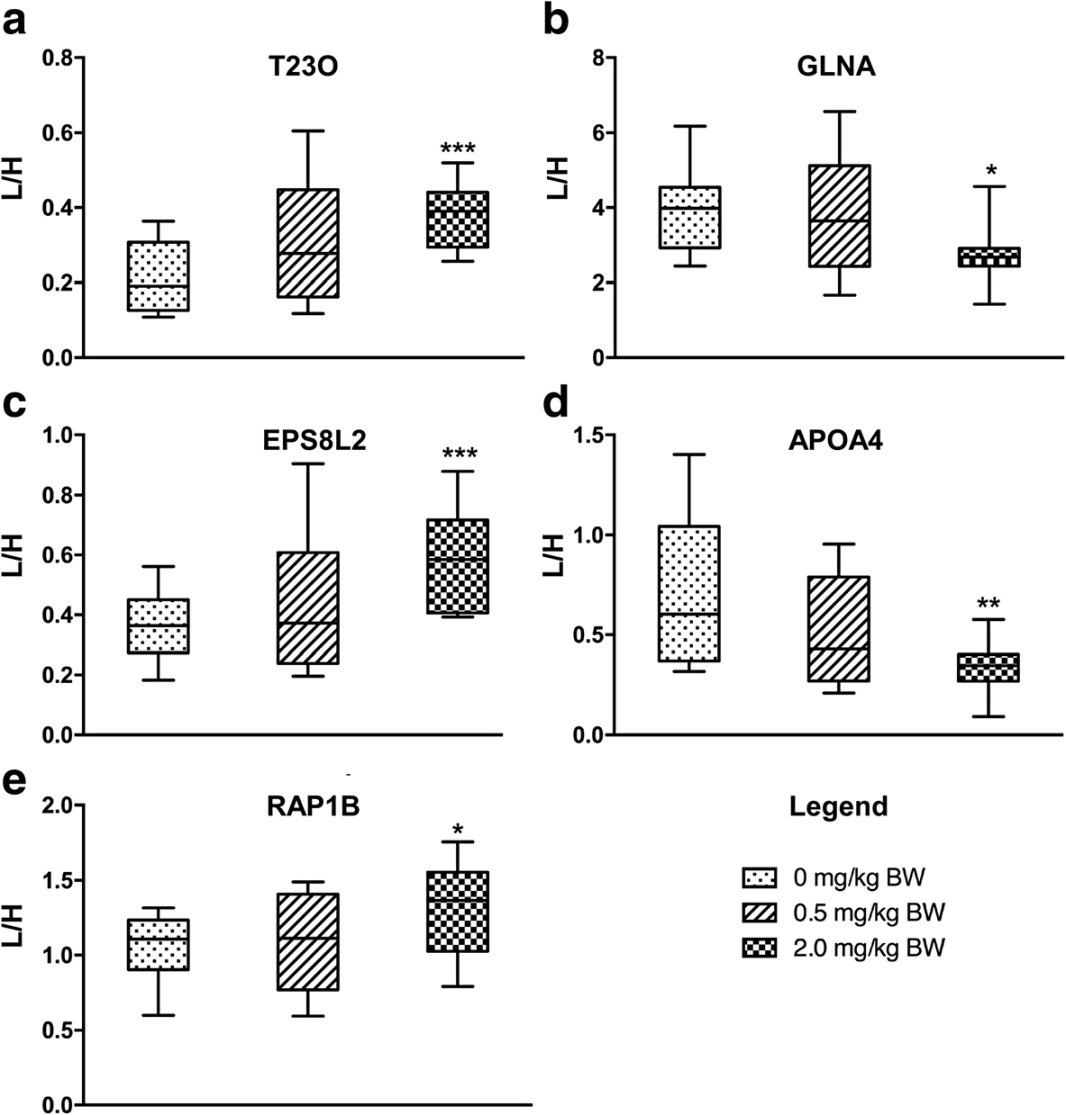
SRM validation of differential protein expression. Peptides corresponding to the indicated proteins (shown here) were quantified using SRM for individual samples in each of control (*n* = 10), 0.5 mg/kg BW MeHg (*n* = 9) and 2 mg/kg BW MeHg (*n* = 9) groups. **a**) T23O; VFVDLFNLATFLIPR. **b**) GLNA; RPSANCDPYAVTEALIR. **c**) EPS8L2; DVQILNCALDDIELLVAR. **d**) APOA4; LTVNTQDLQSQLAELWK. **e**) RAP1B; LQIWDTAGQER. Differences between control and treated groups were compared using Student's *t* test (*p < 0.05, **p < 0.01, and ***p < 0.001). Data are presented as mean ± standard deviation, and the y-axis represents the ratio of intensities between the endogenous peptide (L) and the corresponding isotopically labeled peptide that was added to the sample (H)

The enzymes glutamine synthetase (GLNA) and sodium-potassium ATPase (AT1B1), which decreased in abundance after MeHg exposure (Additional file 2: Table S1), were previously reported to have decreased activity after MeHg-induced oxidative stress in rat brain, and these effects were mitigated by antioxidants [41]. Another enzyme, tryptophan 2,3-dioxygenase (T23O), up-regulated in MeHg-treated samples (also confirmed by SRM) (Additional file 2: Table S1, Fig. 2a), has previously been shown to have increased activity in mercuric chloride treated rat liver [42]. An enzyme in the cysteine and methionine metabolism pathway, S-adenosylmethionine synthase isoform type-2 (METK2) increased its abundance in cod liver after MeHg treatment (Additional file 2: Table S1). METK2 has been shown to be important in oxidative stress responses [43]. Methionine adenosyltransferases have oxidation-sensitive Cys-residues, and they have been shown to be redox regulated [44].

Some of the proteins and enzymes in the cod liver that responded to MeHg exposure have previously been associated with different human diseases. For instance, one of the proteins that increased the most after MeHg exposure, T23O (Additional file 2: Table S1, Fig. 2a) was reported to have increased abundance in the brain of patients with schizophrenia and Alzheimer’s disease (AD) [45, 46]. T23O is a rate limiting-enzyme in the tryptophan degradation pathway, and regulates the levels of the amino acid tryptophan in the body. Apolipoprotein E (ApoE) variants and other cholesterol-related genes have been associated with the pathogenesis of AD [47]. The protein levels of APOB, APOE, APOC1 and APOA4 decreased in the high dose MeHg-treated fish liver (Additional file 2: Table S1). It has been shown that genetic ablation of Apoa4 gene in a mouse model accelerated the development of AD [48]. The differential regulation of the products of the genes previously associated with the AD appears to support the hypothesis that environmental chemicals such as MeHg may contribute to its etiology [49]).

**Fig. 3:**
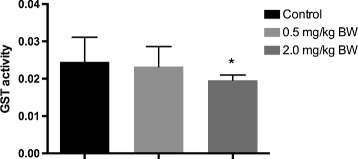
Enzymatic activity of glutathione (GST) in liver homogenates. GST activity was measured in liver homogenate in each of control (*n* = 10), 0.5 mg/kg BW MeHg (*n* = 9) and 2 mg/kg BW MeHg (*n* = 9) groups. Differences between control and treated groups were compared using Student's *t* test (*p < 0.05). Data are expressed as mean (GST activity per min per mg protein) ± standard deviation

Levels of the antioxidant proteins glutaredoxin-1 (GLRX1) and peroxiredoxin-2 (PRDX2), belonging to the glutaredoxin and thioredoxin antioxidant systems, respectively, decreased after MeHg exposure (Additional file 2: Table S1). The decrease in GLRX1 and PRDX2 appears to be in agreement with other studies demonstrating decreased enzymatic activities of the antioxidant enzyme systems caused by MeHg-induced oxidation of their Cys residues [17–19]. PRDX2 is a thiol-specific antioxidant protein known to be modified upon oxidation [50]. All proteins in the GO molecular function “antioxidant activity” (GSTO1, GSTT1, GSTT2, PRDX2, APOE and APOA4) (Table 1B, Additional file 4: Table S3) had lower abundances in the high dose MeHg exposed group (Additional file 2: Table S1). Furthermore, GST activity was measured in the individual liver homogenates using a GST assay, and in line with the proteomics data, the GST activity decreased with increasing MeHg exposure (Fig. 3). Decreased activities of some antioxidant enzymes, including GSTs, have previously been shown to be positively correlated with mercury contamination in fish [51, 52]. Intriguingly, GSTT1 was up-regulated at the mRNA level in the same liver samples [37]. The reason for this discordance is not clear, but it might be due to oxidation-related post-translational modifications and possible degradation of the protein. It is known that oxidatively modified and damaged proteins undergo degradation in the cell [53]. Another thiol oxidation sensitive enzyme, glutamine synthetase (GLNA) (down regulated here) (Fig. 2b), was previously shown to be degraded upon oxidation [54]. The binding of MeHg to thiol groups of proteins (S-mercuration) can lead to their inactivation and aggregation [55]. Indeed, this property has been used to precipitate and identify another protein, sorbitol dehydrogenase (DHSO), which contains reactive Cys residues [56]. DHSO decreased in abundance in cod liver after MeHg treatment (Additional file 2: Table S1). The enzyme GAPDH (G3P) also decreased its abundance in cod liver after MeHg exposure (Additional file 2: Table S1). It has been shown that GAPDH can be oxidized at its active site Cys residue, resulting in misfolding and inactivation [57, 58]. Occurrence of misfolding and conformational changes of proteins was suggested by enrichment of the unfolded protein response (UPR) pathway observed in comprehensive transcriptome analysis of the same samples [37]. Among the up-regulated chaperone transcripts was GRP78 (78 kDa glucose-regulated protein), which is a key regulator of UPR [59]. Activation of UPR can trigger degradation of misfolded proteins and suppression of protein translation [59]. Thus, it is possible that the decreasing levels of some antioxidant enzymes and other proteins observed in this study are related to MeHg-induced conformational changes, possibly resulting in aggregation and degradation. Some studies suggest that an overwhelmed antioxidant defense system may lead to decreased activity of antioxidant enzymes such as GSTs [52, 60].

**Table 1:** The top significantly enriched GO BP, MF and MetaCore “Process Networks” annotations^a^

#	A) GO processes	In data	FDR
1	Small molecule metabolic process	56	2.049E-11
2	Response to inorganic substance	29	4.840E-10
3	Macromolecular complex remodeling	10	4.840E-10
4	Plasma lipoprotein particle remodeling	10	4.840E-10
5	Protein-lipid complex remodeling	10	4.840E-10
6	Cholesterol efflux	10	1.124E-09
7	Glycerolipid catabolic process	11	1.124E-09
8	Single-organism metabolic process	76	1.931E-09
9	Metabolic process	121	1.931E-09
10	Plasma lipoprotein particle organization	10	2.287E-09
#	B) GO Molecular functions	In Data	FDR
1	Oxidoreductase activity	19	7.654E-04
2	Catalytic activity	63	7.654E-04
3	Phosphatidylcholine-sterol O-Acyltransferase activator activity	3	7.654E-04
4	Small molecule binding	37	7.654E-04
5	Cholesterol transporter activity	4	7.654E-04
6	Sterol transporter activity	4	7.654E-04
7	Antioxidant activity	6	7.654E-04
8	Poly(A) RNA binding	21	1.899E-03
9	Anion binding	36	2.555E-03
10	Nucleotide binding	32	2.621E-03
#	C) Process Networks	In Data	FDR
1	Translation_Translation initiation	9	1.001E-02
2	Response to hypoxia and oxidative stress	8	1.999E-02
3	Cytoskeleton_Actin filaments	8	2.223E-02
4	Cell adhesion_Cell junctions	7	4.893E-02

^a^Only the top 10 enriched GO BP (A) and MF (B) are shown here, and the full list of top 50 significantly enriched (FDR < 0.05) GO BP and MF terms and the associated proteins are presented in Additional file 4: Table S3

### Selected reaction monitoring (SRM)

To verify the label-free quantitative proteomics data, six proteins (T23O, GLNA EPS8L2, APOA4, RAP1B, CATZ) among the top differentially regulated were selected and quantified with the more specific SRM method. At least one peptide originating from each of the six selected proteins was quantified using SRM. Peptides from five of the selected proteins (T23O, GLNA EPS8L2, APOA4, RAP1B) showed significant changes in the SRM analyses in agreement with the label-free quantification (Fig. 2a-e, Additional file 2: Table S1). Three other SRM-quantified peptides selected for the APOA4 protein also showed significant decreases (Additional file 1: Figure S3A-C), similar to the 4^th^ peptide shown in Fig. 2d. The peptide for cathepsin Z (CATZ), which was found to have increased levels in MeHg-treated samples with the label-free method (Additional file 2: Table S1), did not change significantly using the SRM method (Additional file 1: Figure S3D). Thus, the SRM data showed largely good concordance with the label-free quantitation, confirming reliability of the quantitative data generated by the latter method.

### Biomarker candidates

Quantitative proteomics studies may help to identify proteins that can be used as biomarkers in mechanistic toxicological studies and environmental pollution monitoring. In this regard, using label-free quantitative proteomics in the discovery phase for identifying altered proteins, followed by verification with SRM, can be a useful method in identification and quantification of potential biomarker proteins. Verification of differentially regulated candidate biomarkers by the SRM method is particularly useful when antibodies are unavailable for quantification of the proteins of interest by immunological methods, such as Western blotting and ELISA.

**Fig. 4:**
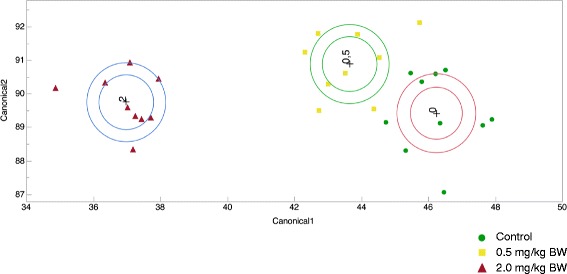
Stepwise discriminant analysis differentially regulated proteins. A set of 8 proteins (five of them confirmed by SRM) correctly classified all 28 individual samples into the three groups. Circles indicate 95 % confidence limits for the group means. Non-intersecting circles indicate significantly different groups

Discriminant analyses using the five proteins verified by SRM in this study were performed in order to investigate their discriminating power between the controls and MeHg-exposed samples. The conjoint expression profile of these five proteins was able to discriminate between the controls and the samples exposed to the highest dose of MeHg (not shown). Furthermore, by using a stepwise variable selection in the discriminant analyses, the addition of three more proteins (FLNA, GTR2, OCM2) was sufficient to also separate the low-dose MeHg samples from the controls without any misclassification (Fig. 4). Some of these eight proteins, and a few others found differentially regulated in the label-free proteomics analyses, may be evaluated as potential biomarkers of hepatic MeHg exposure in future studies. For example, among the 5 proteins validated by SRM, GLNA and T23O have been reported to be modulated by mercury exposure, as mentioned above, although these effects appear to be related to oxidative stress [41, 42], and it is not clear if they are specific to mercury exposure.

A more promising biomarker candidate of particular interest is the enzyme uroporphyrinogen decarboxylase (DCUP/UROD), found down regulated here (Additional file 2: Table S1). UROD is an enzyme in the heme biosynthetic pathway and decreased activity of DCUP has been associated with the human diseases porphyria and liver damage [61]. It has been shown that MeHg inhibits DCUP in rat kidney, leading to increased porphyrin excretion [62]. The urinary porphyrin excretion profile was specific to mercury compounds, and thus it was suggested as a specific biomarker of mercury exposure [62]. Therefore, down regulation of UROD in MeHg treated cod makes it a strong candidate as a biomarker of MeHg exposure. Liver and blood UROD activity and porphyrin profile might be evaluated as biomarkers for MeHg exposure in fish. Down regulation of other proteins involved in heme metabolism such as heme-binding protein 2 (HEBP2) and biliverdin reductase B (BLVRB) (Additional file 2: Table S1) also suggest perturbation of heme homeostasis in MeHg treated cod liver. Consistent with our results, lower liver UROD activities and increased porphyrin levels were detected in pike from River Rhine polluted with halogenated hydrocarbons and heavy metals including mercury [63]. However, UROD activity and porphyrin profiles are not commonly used as biomarkers in fish [64], and our results encourage further potential for development. Aryl hydrocarbon receptor agonist halogenated hydrocarbons have been shown to induce prorphyrin accumulation in the fish hepatoma cell line (PLHC-1) [65]. Thus, in further experiments, changes in expression and activity of UROD combined with porphyrin profile may be performed to evaluate the specificity of responses to mercury exposure in hepatoma cell line [65] and in *ex vivo* systems such as precision-cut liver slices [66].

### Enriched pathways

**Fig. 5:**
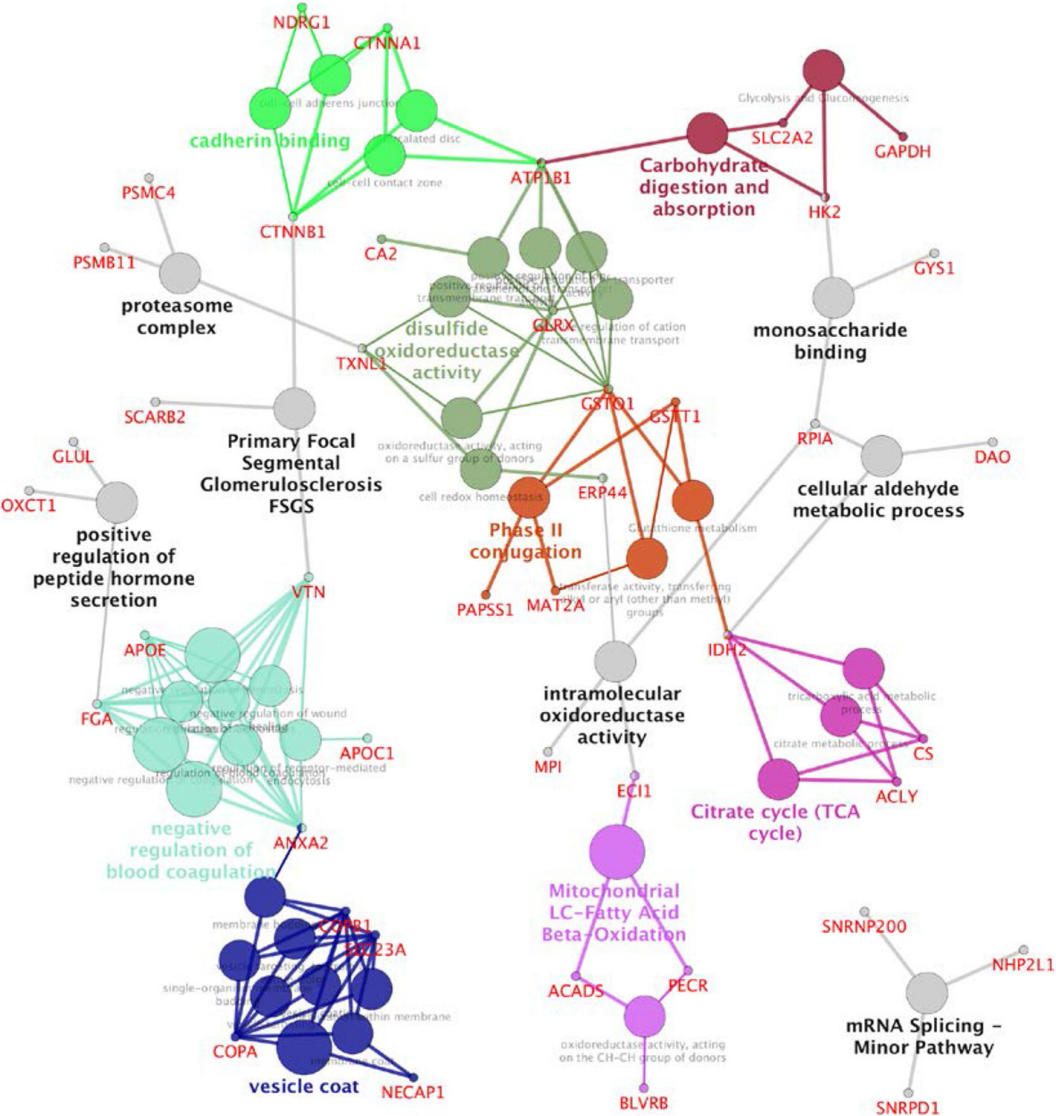
Networks of differentially regulated proteins in MeHg treated samples. The networks were constructed in Cytoscape using CluePedia plug-in. Enrichment analysis was performed using GO (BP and MF), KEGG, REACTOME and WikiPathways at FDR threshold of 0.05. Each group (GO term or pathway) and associated proteins share same color, and node size indicates significance level. For full names of abbreviated of protein names and synonyms, see Additional file 1: Table S1

Annotation and enrichment analysis of the differentially regulated proteins (Additional file 2: Table S1) was performed using MetaCore software and database. GO terms and MetaCore “Process Networks” highlight enrichment of different energy metabolisms (particularly lipid metabolism), antioxidant defense, translation, cytoskeleton remodelling, and cell adhesion related pathways (Table 1A-C, Additional file 4: Table S3). The top GO BP term “small molecule metabolic process” contains proteins in the metabolism of lipids (e.g. PLA2, POA4, APOE), amino acids (e.g. MAT2A, GLNA, TDO2), carbohydrates (e.g. HXK2, RPIA), fatty acids (e.g. PECR, ACDS), and antioxidant defense systems (e.g. GSTs, PRDX2) (Additional file 2: Table S1, Additional file 4: Table S3). Terms #3-7 and 10 in GO BP (Table 1A) are redundant and largely related to plasma proteins. The most significantly enriched GO MF term “oxidoreductase” contains proteins involved in both the antioxidant defense systems (such as GSTs) and the energy pathways, such as ACADS and IDHP (IDH2) (Table 1B, Additional file 4: Table S3). GO MF terms “oxidoreductase activity” and “antioxidant activity” (Table 1B) and process network “response to hypoxia and oxidative stress” (Table 1C) share the antioxidant response proteins GSTO1, GSTT1, GSTT2 and PRDX2 (Additional file 4: Table S3). Similarly proteins in the process network “translation initiation“are a subset of proteins in GO MF term “poly(A) RNA binding” (Table 1C, Additional file 4: Table S3). Many of the enriched pathways (Table 1A-B, Additional file 4: Table S3) are also represented in networks of differentially regulated proteins (Fig. 5, Additional file 1: Figure S4, Additional file 5: Table S4) (see below). Further, protein domain enrichment analysis in STRING also resulted in significant enrichment (FDR < 0.05) of the Interpro protein domain “thioredoxin-like fold”, which is present in the 7 proteins GSTO1, GSTT1, PRDX2, ERP44, TXNL1, GLRX and PRDX2 (Additional file 1: Figure S4), thus supporting MeHg-mediated effects on the antioxidant systems.

Enrichment of the energy pathways supports the effects of MeHg on these cellular processes, particularly mitochondrial metabolic pathways as observed previously by gene expression analysis of the same samples [37]. In the previous gene expression analysis, transcripts of many mitochondrial enzymes, such as acyl-CoA dehydrogenases were particularly affected, suggesting specific effects of MeHg-induced oxidative stress. Recently, Cys-residues of many mitochondrial enzymes have been shown to be oxidized upon Cadmium-induced oxidative stress in mouse liver [40]. Notably, Cys residues of some of the proteins differentially regulated here (ACADS, GLNA, IDHP/IDH2, ERP44, PECR and TRFE) (Additional file 2: Table S1), were found to be differentially oxidized in the Cd-treated mouse liver samples [40]. Among these, ACADS, IDHP/IDH2 and PECR are enzymes that are part of cellular energy pathways. Importantly, ERP44 is a multifunctional chaperone with a Cys-residue that is critical for its function in quality control of proteins in the early secretory pathway, redox homeostasis, and regulation of calcium flux [67]. In our previous transcriptome analysis of the same samples, we also observed differential regulation of several genes participating in different energy pathways, particularly the fatty acid and amino acid metabolic pathways [37]. The similar responses to cadmium chloride and MeHg suggest that differential regulation of the genes or proteins are mediated by induction of oxidative stress by these compounds.

Mitochondrial energy generating pathways (electron-transport chain and fatty acid beta-oxidation) were found to be affected by HgCl_2_ in zebrafish liver [68], which is also in agreement with our results. Similarly, proteins that are part of the antioxidant defense and energy pathways were modulated in Atlantic salmon fed with a MeHg-containing diet [26]. Moreover, energy and redox pathways were also affected in the brain, demonstrated by two-dimensional gel electrophoresis analysis of the brain proteome of Atlantic cod [24] as well as transcriptomics studies of zebrafish [27]. Recently, by proteomics analysis, it was also reported that chronic exposure to low doses of MeHg resulted in down-regulation of energy pathway proteins and metabolic deficit in the somatosensory cortex of the rat brain [22]. In another recent proteomics study of the brain of common marmosets, proteins involved in the metabolism of carbohydrate derivatives were found to be down regulated, suggesting negative effects of MeHg on the carbohydrate metabolism [23]. Taken together, these results strongly suggest that oxidative stress mediated effects on energy pathways is a general mechanism of MeHg-mediated toxicity.

### Comparison of proteomics and gene expression data

Comparison of significantly enriched (FDR < 0.05) top 50 annotations (GO BP, MF and MetaCore process networks) between the trancriptomics [37] and proteomics data obtained from the same cod liver samples showed many shared pathways (Additional file 1: Figure S5). As expected, a higher number of genes (650 genes) were differentially regulated in the microarray analysis compared to the identified number of differentially regulated proteins (125 proteins). However, twenty-six annotations were shared between the two datasets. The main processes previously known to be affected by MeHg, such as energy metabolism, oxidoreductase activities, protein translation, and cytoskeleton remodelling, were enriched in both datasets, showing agreement between the two methods.

Although analysis of transcriptomics and proteomics data revealed many shared pathways, only 11 genes were present in both lists of differentially regulated genes and proteins. Of these 11 genes, 6 showed concordance in expression changes at mRNA and protein levels (genes encoding ACADS, METK2, SCOT1, EPS8L2 and FUBP2 increased, while CATZ decreased). In contrast, genes encoding IDHP/IDH2, GSTT1, RL19 and TRI16 increased at the mRNA levels and decreased at the protein levels. The gene encoding LPP60 decreased at mRNA level and increased at protein level. Thus, changes in 5 of the 11 proteins were not consistent with changes in mRNA levels. The overall correlation for 32 common transcripts/proteins detected at more relaxed differential regulation cut-offs for transcripts (FDR < 5 % and minimum 1.2 fold change) and proteins (*p* < 0.05) was low (*r* = 0.16), with only 14 of them changing in the same direction (not shown). The lack of strong correlation between transcript and protein changes is not surprising. Numerous studies in various organisms reported low to modest correlation between mRNA and protein levels [69–71]. In mouse liver, not more than 50 % of the genes showed a significant correlation of 0.27 between transcript and protein levels [69]. In a study comparing transcript and protein expression responses to a toxicant (2A-DNT) treatment, poor correlation was observed, although higher order pathways affected were similar [70]. Similarly, in our study proteomics and transcriptomics datasets shared many GO annotations despite low correlation at the individual transcript and protein expression levels (Additional file 1: Figure S5). Factors that may contribute to the low correlation may be post-translational modifications, localization (ex. secreted proteins), and different dynamics of mRNA and protein turnover. As discussed above, degradation of some proteins triggered by MeHg-induced oxidation could be one factor contributing to low correlation between protein and mRNA levels.

To further investigate integrated effects of MeHg in the cod liver, we combined the differentially regulated 650 genes and the 125 proteins and performed network analysis and enrichment of GO (BP and MF), KEGG, Wikipathways, and REACTOME pathways using the CluePedia application in Cytoscape. The generated network highlights major processes and pathways affected, including amino-acid metabolism, fatty-acid metabolism, glucose metabolism and protein synthesis (Additional file 1: Figure S6). The energy pathways dominate in the combined dataset due to the high number of energy pathway genes, particularly amino acid and fatty acid metabolism genes represented in the larger transcriptomics dataset [37]. Processes and pathways mostly related to oxidative stress such as sulphur compound metabolism, glutathione metabolism and oxidation and reduction processes, and co-factor metabolism, are also highlighted (Additional file 1: Figure S6). Notably, the pentose phosphate pathway (PPP) that generates NADPH required as electron donor in the antioxidant systems is significantly enriched in the combined dataset (Additional file 1: Figure S6).

Finally, comparison of GO BP and MF affected in MeHg treated cod as determined by liver transcriptomics analysis [37], liver proteomics (this study) and brain proteomics (same fish as used in this study) [24] shows that processes related to energy metabolism and oxidative responses are shared among the three studies (Additional file 1: Figure S7). For example, among the 12 significantly enriched GO terms shared between the datasets, “small molecule metabolic process” and “oxidoreductase activity” are related to energy and redox pathways (Additional file 1: Figure S7).

Other enriched pathways include pathways related to translation, cytoskeleton, vesicle transport and cholesterol transport (Table 1; Additional file 4: Table S3, Additional file 1: Figure S4). Effects on protein synthesis and the cytoskeleton were also observed in both transcriptomics and proteomics analyses (Additional file 4: Table S3, Additional file 1: Figure S4). It is not clear if some of the enriched pathways are activated or suppressed from our analysis as the differential regulation of component proteins largely changed in both directions (Additional file 1: Figure S4). For example, some proteins involved in poly(A) RNA binding related to translation increased and others decreased, and the outcome on protein synthesis is not clear. However, previous studies have reported that MeHg inhibits protein synthesis [5]. The enrichment of the cytoskeletal assembly pathway is possibly related to the known negative effect of MeHg on microtubule assembly [15]. Tubulin alpha and beta chains were also among the polypeptides with reactive thiol groups identified in the liver thiol proteome analysis of the same cod liver samples [25].

### Network analyses suggest possible interactions of oxidative stress and energy pathways

Network analysis of the differentially regulated proteins was performed in Cytoscape (http://www.cytoscape.org/) using the Cluepedia plug-in [72]. Similar to the GO terms and process networks enriched in the MetaCore analysis (Table 1, Additional file 4: Table S3), the generated networks highlighted energy pathways (e.g. TCA cycle and fatty acid beta oxidation) and antioxidant responses (e.g. phase II conjugation) (Fig. 5). Other pathways related to the significantly enriched GO terms “vesicle-mediated transport” and “blood coagulation” and “cadherin binding” (Additional file 4: Table S3) can also be visualized in the Cytoscape network (Fig. 5).

Furthermore, protein-protein interaction network analysis of the differentially regulated proteins was performed using the STRING database and web-tool (http://string-db.org). STRING also enables enrichment analysis and visualization of KEGG, GO and protein domains in protein networks. Annotations consisting of 6 KEGG pathways, 9 GO BP terms, 2 GO MF terms, 16 GO CC terms and 1 Interpro protein domain were significantly enriched at FDR *p*-value < 0.05 (Additional file 1: Figure S4, Additional file 5: Table S4).

The networks suggest interesting interactions of proteins and pathways of energy metabolism and antioxidant systems. Notably, the networks show separate clustering of energy pathway (TCA cycle) proteins and antioxidant response proteins (disulfide oxido-reductase activity, phase II conjugation) linked by the mitochondrial isocitrate dehydrogenase enzyme (IDPH/IDH2) node (Fig. 5). IDH2, that was down regulated in MeHg exposed samples (Additional file 2: Table S1), is an energy pathway enzyme that is also a key component of the antioxidant defense, being important in the NADPH generation in the mitochondria [73]. NADPH is important in providing reducing potential to the glutaredoxin and thioredoxin antioxidant systems. Although the consequence of IDH2 down regulation on energy pathways is unclear from our results, mitochondrial IDH2 function is known to be influenced by ROS [74], and it has been suggested that its activity is specifically modulated through oxidation of an important Cys-residue upon oxidative stress [75]. Consistent with these previous reports, our network analysis suggests that IDH2 could be one of the links for interactions between antioxidant systems and energy pathways (Fig. 5). Indeed, recent studies have shown that ROS levels can regulate the activities of enzymes in the mitochondrial energy generating pathways through Cys thiol modifications [76].

Further, STRING protein-protein interaction networks and enrichment analysis also suggest crosstalk between energy pathways and redox homeostasis (Additional file 1: Figure S4). For example, ATP-citrate lyase (ACLY) that is known to be redox-regulated [77], forms an important hub in STRING protein-protein interaction network linked with many energy pathway and antioxidant proteins (Additional file 1: Figure S4). Another hub is formed by GAPDH, which is a glycolytic enzyme known to be involved in antioxidant responses [78]. ACLY, GAPDH and many energy pathway enzymes (e.g. TCA cycle and fructose and mannose metabolism) were down regulated in MeHg treated samples, suggesting suppression of the cellular energy pathways (Additional file 1: Figure S4, Additional file 2: Table S1). Suppression of the energy pathways is consistent with presence of reactive Cys residues in many of the energy pathway enzymes discussed above that might make them targets of MeHg-indued oxidation. Importantly, it has been reported that oxidation of GAPDH enables the re-routing of metabolic flux from glycolysis to the Pentose Phosphate Pathway (PPP) in order to generate NADPH required for the antioxidant systems [78]. This is another example of redox modulation of an energy pathway. The PPP is one of the significantly enriched carbohydrate metabolism pathways in the combined transcriptomics and proteomics dataset (Additional file 1: Figure S6), suggesting its activation in response to increased oxidative stress. These results provide some mechanistic insights into how MeHg-induced oxidative stress might be disrupting cellular redox balance and energy metabolism.

Recent studies have identified oxidation sensitive Cys residues in many mitochondrial enzymes and suggest modulatory effects of oxidative stress on the energy metabolism [40, 79], which is consistent with our results. It has been suggested that the interaction of redox and metabolic pathways are coordinated by the transcription factor nuclear factor erythroid 2-related factor (Nrf2), which regulates transcription of genes in phase II and phase III detoxification pathways, the antioxidant glutaredoxin and thioredoxin systems, and energy pathways, including carbohydrate metabolism and NADPH generation [80]. In fact, many Nrf2 regulated genes were regulated in our MeHg treated samples, and the Nrf2 regulatory pathway was significantly enriched in the transcriptomics analysis [37]. Long established effects of MeHg, such as mitochondrial dysfunction, altered calcium homeostasis, and apoptosis, are thought to be consequences of its oxidative stress promoting characteristics [12, 13]. Although other studies have reported effects of MeHg on energy pathways [22, 23, 27, 81], our integrated transcriptomics and proteomics data indicate more comprehensive effects of MeHg on major energy pathways, in particular the mitochondrial fatty acid metabolism. The apparently more coordinated effects observed on energy metabolic pathways in our studies, perhaps reflect the extent of the data (integrated transcriptomics and proteomics) and the fact that the main physiological role of the liver is metabolism. The overall results indicate that oxidative stress may lead to perturbations in energy pathways in cod liver, further supporting induction of oxidative stress as the underlying mechanism of cytotoxic effects of MeHg. Although oxidation of proteins is often reversible, persistent exposure to MeHg may lead to irreversible oxidation of thiol groups causing enzyme inactivation, mitochondrial dysfunction and other cellular damages.

## Conclusions

Extensive proteomics analysis of liver samples from cod treated with MeHg indicated oxidative stress responses and perturbation of cellular energy pathways, confirming our earlier findings by transcriptomics analysis. The integrated analyses with both transcriptomics and proteomics data, together with previous studies, further support that MeHg-induced oxidative stress modulates cellular energy pathways, contributing to well-known toxic effects of this compound, such as mitochondrial dysfunction and other cellular damages.

## Methods

### Fish exposure and sampling

Fish exposure and sampling has been described previously [24]. Briefly, juvenile Atlantic cod (*G. morhua*) of mixed gender (body weight of 260–530 g) were divided into four 500 L-tanks for control, 0.5, 2 and 8 mg/kg groups (*n* = 10/group), and acclimated for 6 days in continuously running seawater (temperature 10 ° C and salinity 34‰) at the facility in ILAB, Bergen, Norway. The fish were fed daily with commercial pellets (Ewos marin 6 mm, EWOS, Bergen, Norway). Methylmercury chloride (CH_3_ HgCl) (Strem Chemicals, Newburyport, USA) was dissolved in the vehicle (20 % acetone and 80 % soybean oil). The vehicle (control), 0.5, 2 or 8 mg/kg body weight methylmercury chloride was injected intraperitoneally by inserting the needle carefully into the abdominal cavity, not to puncture internal organs. The indicated doses were divided into two and the first half injected on day 1, and the second half after one week. After the second injection all the fish in the highest (8 mg) dose group and 1 fish from each of the 0.5 and 2 mg dose groups died. The fish were observed and behavioural parameters such as overall activity and balance was registered during the exposure period. After 2 weeks (14 days) the fish were sacrificed by decapitation and tissue samples collected. Liver samples were dissected, weighed and snap frozen in liquid nitrogen and stored at −80 °C. The National Animal Research Authority approved the exposure experiment. Fish length (in cm) and weight (in g) were recorded at the start and end of the experiment. Condition factor (100 * weight/length^3^) and liver-somatic index (100 * liver weight/BW) were calculated, and Student's *t*-test was used to calculate *p*-value between sample groups.

### Sample preparation

Homogenization of liver samples has been described previously [25]. Briefly, frozen individual cod liver sample obtained from each of control (*n* = 10), 0.5 mg/kg BW MeHg (*n* = 9) and 2 mg/kg BW MeHg (*n* = 9) groups (from 28 fish in total) was thawed on ice, washed and homogenized in phosphate-buffered saline (PBS, pH 7.4). The homogenate was centrifuged at 13,000 × g for 10 min and the supernatant containing hepatic proteins was digested with trypsin, and the resulting peptides purified as described previously [82].

### Label-free proteomics using LC-MS

The peptide sample (1 μg) from each of control (*n* = 10), 0.5 mg/kg BW MeHg (*n* = 9) and 2 mg/kg BW MeHg (*n* = 9) groups was dissolved in 1 % aqueous formic acid (FA) and injected into an Ultimate 3000 RSLC system (Thermo Scientific) connected online to an LTQ-Orbitrap Velos Pro mass spectrometer (Thermo Scientific) equipped with a nanospray Flex ion source (Thermo Scientific). The peptides were separated using a 90 min HPLC gradient with increasing acetonitrile concentration in 0.1 % FA and the acquisition time for LC-MS data was 80 min as previously described [82]. The LTQ-Orbitrap Velos Pro settings were the same as previously described [82] except that the normalized collision energy was set to 35 % and the dynamic exclusion to 30s.

### Analysis of the label-free data using Progenesis LC-MS

Progenesis LC-MS® v4.1 (Nonlinear Dynamics Ltd) was used for label-free quantification of automatically aligned LC-MS data. Only peptide features with charges between +2 to +5 containing the top 10 ranked MSMS spectra (with precursor intensities more than 0) and where MSMS was executed on the highest precursor isotopes (less than 6) were accepted. The MSMS spectra list was sorted by rank and the fragment ions in the MSMS spectra were limited to 200 allowing deisotoping and charge deconvolution prior to export as an mgf file for identification. The mgf file was searched using SearchGUI v1.18.1 [83] using the option “Delete duplicate spectra titles”. The search engines X!Tandem, MS Amanda and MS-GF+ Beta were used for searches in the Ensembl *Gadus morhua* protein database. Protein identification was conducted against a concatenated target/decoy version of a complement of the database and the decoy sequences were created by reversing the target sequences in SearchGUI. The identification settings were: trypsin with a maximum of 1 missed cleavages; 10 ppm as MS1 and 0.5 Da as MS2 tolerances; carbamidomethyl Cys as fixed modification; oxidation of Met as variable modification.

Peptides and proteins were inferred from the spectrum identification results using PeptideShaker v0.28 [84]. Peptide Spectrum Matches (PSMs), peptides and proteins were validated at a 1 % False Discovery Rate (FDR) estimated using the decoy hit distribution. The results were exported as validated PSMs and imported into Progenesis. The sum of the normalized abundances of 1143 quantified unique proteins was exported from Progenesis and analyzed further using statistical analyses.

### Proteomics data analysis

For functional annotation and pathway analysis, the 1143 unique cod proteins quantified (Additional file 3: Table S2) were mapped to orthologs of human proteins in Swissprot database using the BioMart tool in Ensembl database (Ensembl.org) or BLAST searches in Swissprot database using the cod protein sequences. There are 22154 genes in the genome of Atlantic cod in the Ensembl database (Ensembl.org). Differential expression analysis was performed using one-way ANOVA followed by Dunnett´s test (JMP, SAS Institute, Cary NC, USA). In addition, we performed differential expression analysis using SAM (Significance Analysis of Microarrays) implemented in J-Express [85] (http://www.molmine.com/) that enables estimation of False Discovery Rates (FDR) in pairwise comparisons. Proteins with *p* value < 0.05 (Dunnett´s test) and FDR < 0.1 (SAM) satisfying a fold change (treated/ control) greater than 1.2 or less than 0.8 were considered differentially regulated. (Additional file 2: Table S1). Although minimum fold change of 1.3-2 is commonly used as cut-off for differential regulation in quantitative proteomics [86], a minimum fold change of 1.2 was used here, considering a relatively large number of biological replicates analyzed (*n* = 9–10 per group). Following these criteria, a total of 125 proteins were identified as significantly differentially regulated between the 2 mg/ kg BW MeHg dose and control groups, of which 44 were up-regulated and 81 were down regulated between controls and 2 mg/kg BW MeHg treated samples (Additional file 2: Table S1). Among the 125 proteins, 7 were differentially regulated in both 0.5 mg and 2 mg/kg BW MeHg dose groups (Additional file 2: Table S1) using the statistical criteria stated above. No proteins were differentially regulated exclusively in the low dose (0.5 mg/ kg BW) group. Hierarchical clustering (complete-linkage) and principal component analysis (PCA) of proteins differentially regulated by MeHg was performed based on log2-transformed abundance ratio values and arcSinh normalised protein abundances, respectively (JMP 11, SAS Institute, Cary NC, USA). Discriminant analyses were performed on arcSinh normalised protein abundances using the Linear method and stepwise variable selection in JMP 11.

### Selected reacting monitoring (SRM)

SRM was performed for six proteins (T23O, GLNA EPS8L2, APOA4, RAP1B, CATZ) among the top differentially regulated. As certain criteria, such as peptide length, protease miscleavage, and amino acid composition must be considered when selecting peptides for SRM analyses, it was not possible to choose freely from the list of proteins/peptides quantified with the label-free approach. Stable isotope –labelled synthetic peptides (SIS) were purchased in crude quality from Thermo Scientific. The C-terminal lysine or arginine in the SIS peptides were replaced by isotope-labelled lysine (^13^C_6_, ^15^N_2_) or arginine (^13^C_6_, ^15^N_4_), resulting in a mass difference of 8 Da and 10 Da, respectively, to the corresponding endogenous peptide. The SIS peptides were spiked in equal amounts into the digested protein samples, at approximately the same level as the endogens peptide, prior to desalting with Oasis HLB μElution Plate (Waters). A Q-Trap 5500 (AB SCIEX) connected to a Dionex Ultimate NCR-3500RS LC system was used for the LC SRM analyses. The protein digest were dissolved in 2 % ACN, 0.1%FA (Buffer A) and 1 μg peptides was trapped on the pre-column (Dionex, Acclaim PepMap 100, 2 cm x 75 μm i.d, 3 μm C18 beads) in buffer A at a flowrate of 5 μl/min for 5 min before separation by reverse phase chromatography (Dionex, Acclaim PepMap 100, 15 cm x 75 μm i.d., 3 μm C18 beads) at a flow of 250 nL/min. The nano LC gradient consisted of a linear gradient starting at 5 % of 90 % ACN, 0.1 % FA (buffer B) and ramping to 40 % buffer B over 45 min (0–45 min). In half a minute the gradient was ramped to 90 % buffer B (45–45.5 min) and held for 6 min (45.5-51.5 min.) followed by ramping to 5 % buffer B for 3.5 min (51.5-55 min) and equilibration of the column in 15 min (55–70 min). The Collision energy (CE) for each peptide was calculated according to the following formulas; CE = 0.044 x m/z +10 for doubly charged precursors and CE = 0.05 x m/z + 9 for triply precursors. The SRM data was analyzed using Skyline v1.4 [87] using the most abundant transition for quantification. Student's *t*-test was used to calculate the *p*-value between sample groups.

### Glutathione-S-transferase (GST) assay

GST activity was determined according to a previously published method [88]. GST activity per min per mg protein was calculated for each sample in control (*n* = 10), 0.5 mg/kg BW MeHg (*n* = 9) and 2 mg/kg BW MeHg (*n* = 9) groups. Treated and control groups were compared using Student’s *t*-test.

### Annotation, pathway and gene ontology enrichment analysis

Enrichment analyses for MetaCore “Process Networks” and GO (Biological Processes and Molecular Functions) were performed in MetaCore™ (MetaCore™; GeneGo-Thomson Reuters, St. Joseph, MI). A false discovery rate (FDR) < 0.05 was used as the threshold for significant enrichment.

### Network analysis, enrichment and visualization

Gene ontology pathway enrichment analysis and network visualization was performed using Cytoscape v3.2.1 (http://www.cytoscape.org/) with the plug-ins ClueGo v2.1.7 [89] and CluPedia v1.1.7 [72], at FDR < 0.05. GO (BP and MF) and KEGG, REACTOME and WikiPathways databases were used. Protein-protein interaction network and KEGG pathway, GO and protein domain enrichment analyses were performed in STRING v10 [90].

## Abbreviations

ACADS, Short-chain specific acyl-CoA dehydrogenase, mitochondrial; ACLY, ATP citrate lyase; APOA4, Apolipoprotein A4; AT1B1, ATPase, Na+/K+ transporting, beta 1 polypeptide; BP, biological process; BW, Body weight; CATZ, Cathepsin Z; CC, cellular component; DHSO, sorbitol dehydrogenase; EPS8L2, Epidermal growth factor receptor kinase substrate 8-like protein 2; ERP44, Endoplasmic reticulum resident protein 44; FDR, false discovery rate; FUBP2, Far upstream element-binding protein 2; GAPDH (G3P), glyceraldehyde-3-phosphate dehydrogenase; GAPDH; GSTO1, glutathione S-transferase omega-1: GLNA, glutamine synthetase; GLNA, glutamine synthetase; GLRX, Glutaredoxin-1; GO, Gene Ontology; GRP78, 78 kDa glucose-regulated protein; GSTT1, glutathione S-transferase theta-1; GSTT2, glutathione S-transferase theta-2B; HXK2, Hexokinase-2; IDHP (IDH2), Isocitrate dehydrogenase [NADP], mitochondrial; KEGG, Kyoto Encyclopedia of Genes and Genomes; LPP60, 60 kDa lysophospholipase; MeHg, methylmercury; METK2:, methionine adenosyltransferase II, alpha; MF; molecular function; NADPH, Nicotinamide adenine dinucleotide phosphate; Nrf2, nuclear factor erythroid 2-related factor; PECR, Peroxisomal trans-2-enoyl-CoA reductase; PPP, Pentose Phosphate Pathway; PRDX2, Peroxiredoxin-2; RL19, 60S Ribosomal Protein L19; RPIA, Ribose-5-phosphate isomerase; SCOT1, Succinyl-CoA; SRM, Selected reacting monitoring; T23O (TDO2), tryptophan 2,3 dioxygenase; TRFE, Serotransferrin; TRI16, Tripartite motif-containing protein 16; TXNL1, Thioredoxin-like protein 1; UPR, Unfolded Protein Response.

## Additional files

Additional file 1:
**Figure S1.** Body weight (A), condition factor (B) and liver-somatic index (C) in control (Ctr), 0.5 mg/kg BW MeHg (0.5 mg) and 2 mg/kg BW MeHg (2 mg) treated Atlantic cod; **Figure S2.** Histograms illustrating the label-free quantification data. Average abundance of each group minus respective median values was log_2_ -transformed and plotted for the control (black line), 0.5 mg/kg BW MeHg (red line) and 2 mg/kg BW MeHg (green line) group (Graph Pad Prism using auto bin range and width); **Figure S3.** SRM-quantification of differential expression for the proteins APOA4, represented by three peptides (A-C), and CATZ (D); **Figure S4** - STRING protein-protein interaction network of differentially regulated proteins; **Figure S5.** Venn diagram showing shared annotations between proteomics and transcriptomics datasets; **Figure S6.** Networks of combined differentially regulated genes identified in transcriptomics and proteomics analyses in MeHg treated samples; **Figure S7.** Venn diagram showing shared annotations among liver proteomics, liver transcriptomics and brain proteomics datasets. (DOCX 11788 kb) Additional file 2: Table S1. 125 proteins differentially regulated by MeHg. (XLSX 94 kb) Additional file 3: Table S2. Full list of 1143 quantified proteins and associated data from label-free proteomics analysis of liver protein samples from controls (*n* = 10), 0.5 mg/kg BW MeHg (*n* = 9) and 2 mg/kg BW MeHg (*n* = 9) fish. (XLSX 610 kb) Additional file 4: Table S3. Full list of significantly enriched top 50 GO BP and MF and all significant MetaCore processes network terms and the associated proteins. (XLSX 73 kb) Additional file 5: Table S4. Full list of significantly enriched KEGG, GO (BP, MF and CC) and Interpro protein domains from STRING network analysis. (XLS 50 kb)
