# Supplementary material for: Quantitative analyses of the hepatic proteome of methylmercury-exposed Atlantic cod (Gadus morhua) suggest oxidative stress-mediated effects on cellular energy metabolism
Source: BMC Genomics. 2016 Aug 5;17:554. doi: 10.1186/s12864-016-2864-2 (PMC4974784; doi:10.1186/s12864-016-2864-2)
Supplement: Additional file 1: — Figure S1. Body weight (A), condition factor (B) and liver-somatic index (C) in control (Ctr), 0.5 mg/kg BW MeHg (0.5 mg) and 2 mg/kg BW MeHg (2 mg) treated Atlantic cod; Figure S2. Histograms illustrating the label-free quantification data. Average abundance of each group minus respective median values was log2 -transformed and plotted for the control (black line), 0.5 mg/kg BW MeHg (red line) and 2 mg/kg BW MeHg (green line) group (Graph Pad Prism using auto bin range and width); Figure S3. SRM-quantification of differential expression for the proteins APOA4, represented by three peptides (A-C), and CATZ (D); Figure S4 - STRING protein-protein interaction network of differentially regulated proteins; Figure S5. Venn diagram showing shared annotations between proteomics and transcriptomics datasets; Figure S6. Networks of combined differentially regulated genes identified in transcriptomics and proteomics analyses in MeHg treated samples; Figure S7. Venn diagram showing shared annotations among liver proteomics, liver transcriptomics and brain proteomics datasets. (DOCX 11788 kb) [file 12864_2016_2864_MOESM1_ESM.docx]

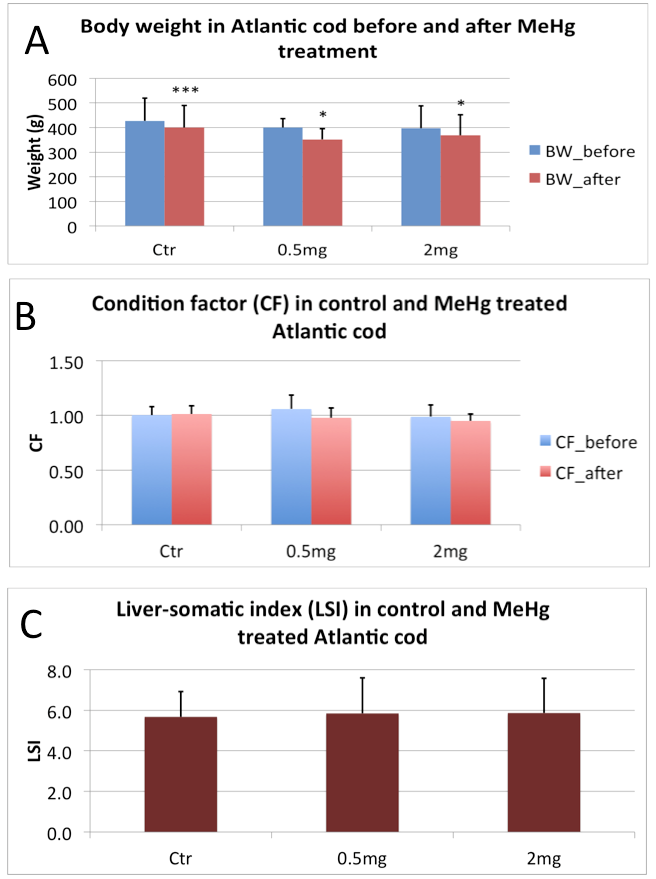


**Figure S1**. Body weight (A), condition factor (B) and liver-somatic index (C) in control (Ctr), 0.5 mg/kg BW MeHg (0.5mg) and 2 mg/kg BW MeHg (2mg) treated Atlantic cod. The designations ”befor” and ”after” indicate values at the start and end of exposure experiments, respectively. Data are presented as mean ± standard deviation. Differences between control and treated groups were compared using Student's t test (**p* < 0.05, ****p* < 0.001).


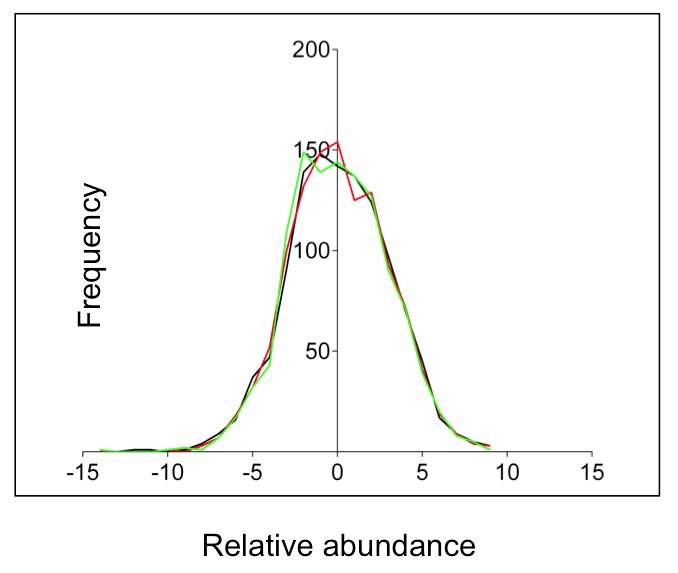


**Figure S2**. Histograms illustrating the label-free quantification data.

Average abundance of each group minus respective median values was log2 -transformed and plotted for the control (black line), 0.5 mg/kg BW MeHg (red line) and 2 mg/kg BW MeHg (green line) group (Graph Pad Prism using auto bin range and width).


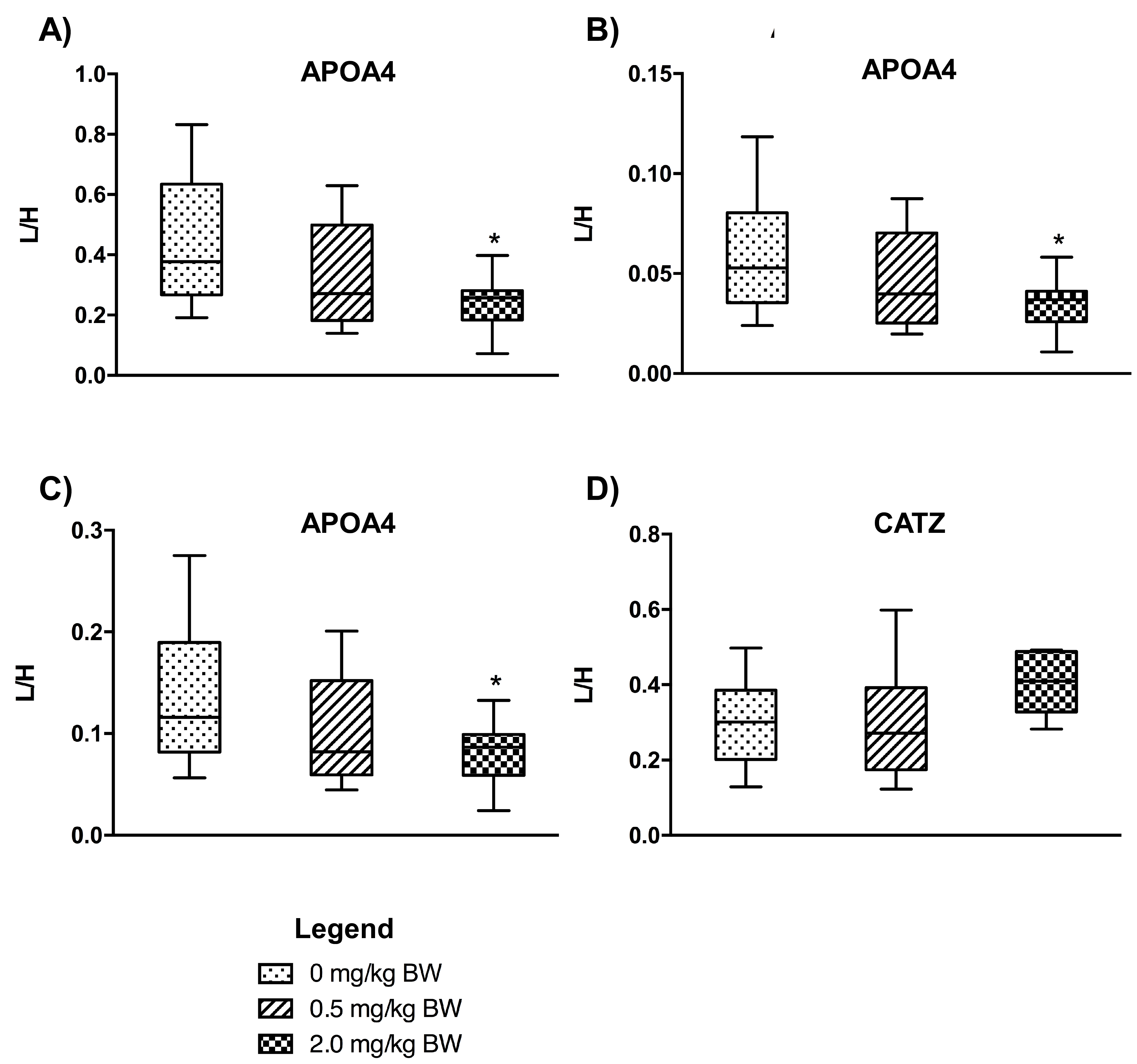


**Figure S3**. SRM-quantification of differential expression for the proteins APOA4, represented by three peptides (**A-C**), and CATZ (**D**). Peptides corresponding to the indicated proteins were quantified using SRM in each of control (n=10), 0.5 mg/kg BW MeHg (n= 9) and 2 mg/kg BW MeHg (n= 9) groups. Differences between control and treated groups were compared using Student's t test (**p* < 0.05). Data are presented as mean ± standard deviation, and the y-axis repr esent the ratios of intensities between the endogeous peptide (L) and the corresponding isotopically labeled peptide that were added to the sample (H).


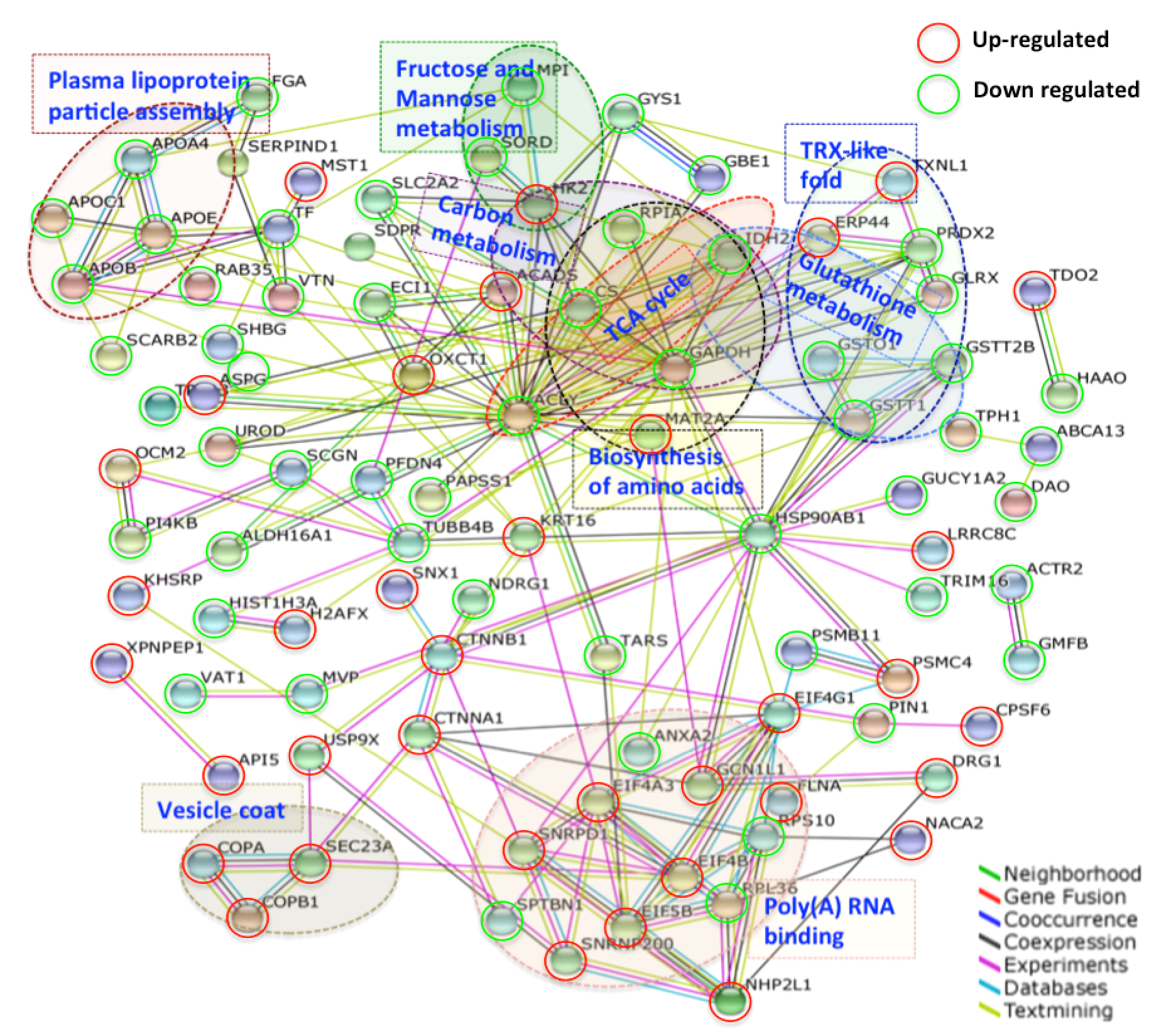


Figure S4 - STRING protein-protein interaction network of differentially regulated proteins. The network is shown in “evidence view” mode, with evidence type shown in color-coded lines (edges) as shown in the legend (bottom right corner). Red and green circles around individual proteins shows up-regulation and down regulation, respectively. Sub networks with well clustered smaller number of nodes (proteins) of significantly enriched (FDR p-value < 0.05) KEGG pathways (“carbon metabolism”, “biosynthesis of amino acids”, “glutathione metabolism”, “TCA cycle” and “fructose and mannose metabolism”), GO BP term “protein-lipid complex assembly”, GO MF term “Poly(A) RNA binding“, GO CC term “Vesicle coat”, and the Interpro protein domain “Thioredoxin (TRX)-like fold “ are manually highlighted as shown, to enhance visualization. Disconnected protein nodes were removed from the networks. Other significantly enriched KEGG pathway and GO terms that are not indicated in this networks are found in the full list of significantly enriched annotations (Additional file 5: Table S4).


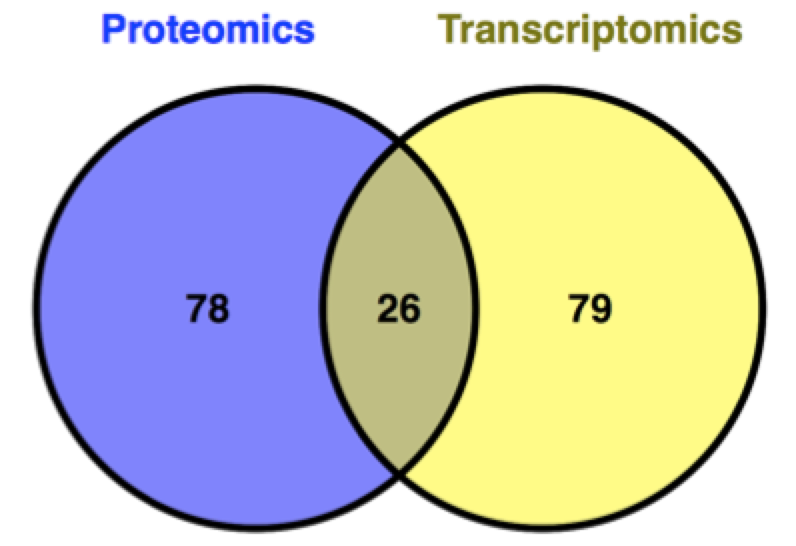

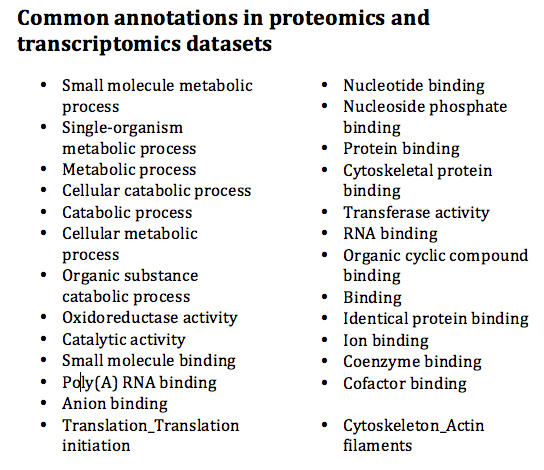


**Figure S5.** Venn diagram (upper panel) showing shared annotations (lower panel) between proteomics and transcriptomics data set. Significantly enriched (FDR < 0.05) annotations (GO BP, MF and MetaCore process network) of differentially regulated 125 proteins in this experiment and 650 differentially regulated genes in the same samples as determined by microarray analysis (Yadetie et al., 2013) were compared, showing 26 common annotations.


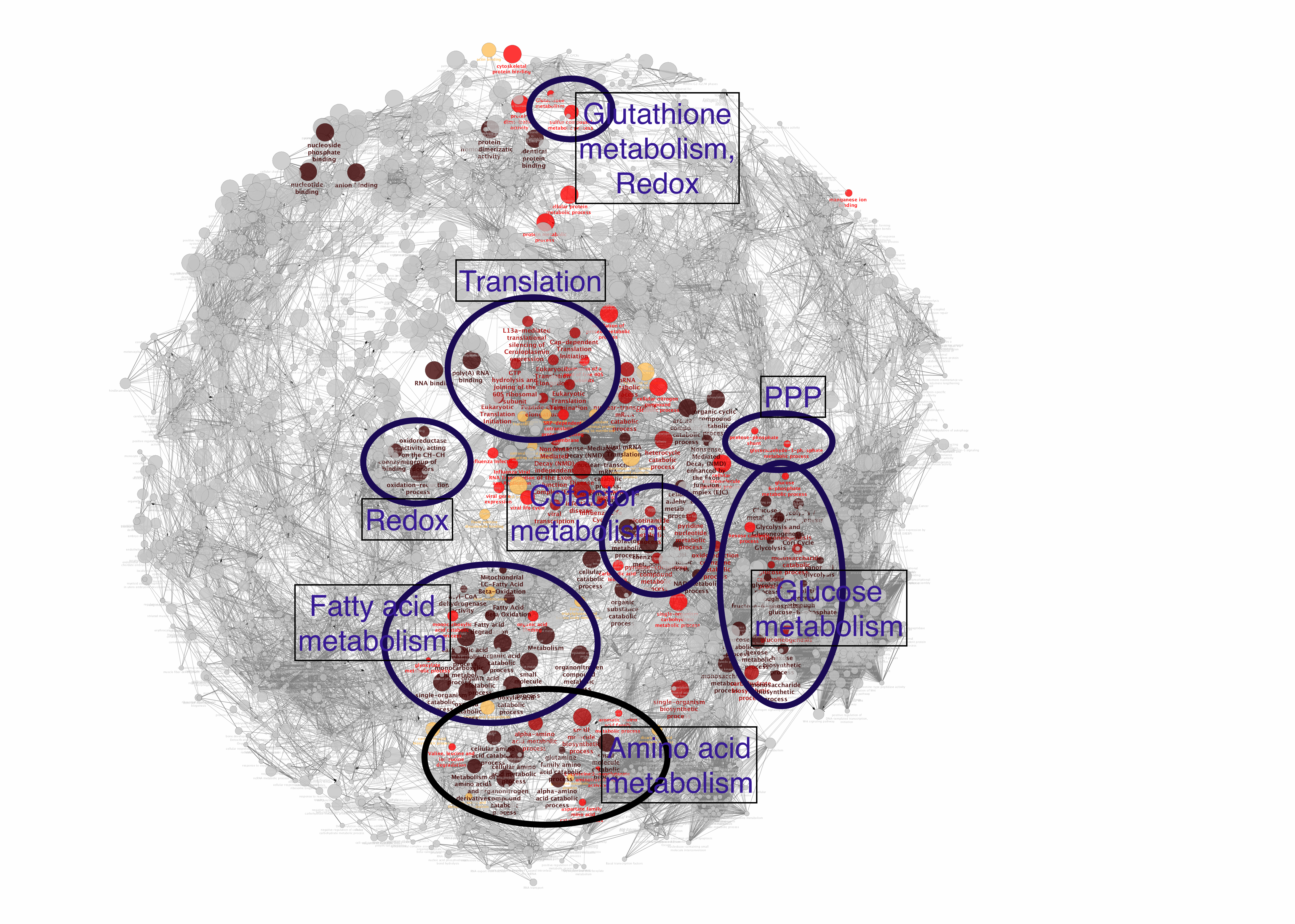


**Legend:**


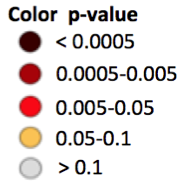


**Figure S6.** Networks of combined differentially regulated genes identified in transcriptomics (Yadetie et al., 2013) and proteomics (this study) analysis in MeHg treated samples. The networks were constructed in Cytoscape using CluePedia plug-in. Enrichment analysis was performed using GO (BP and MF), KEGG, REACTOME and WikiPathways at FDR threshold of 0.05. Node (GO term or pathway) size is proportional to number of mapped genes and color indicates significance level as shown in the legend (bottom right corner). Approximate clusters of major pathways were manually highlighted and labeled, to help visualization. Abbreviation: PPP: Pentose phosphate pathway.


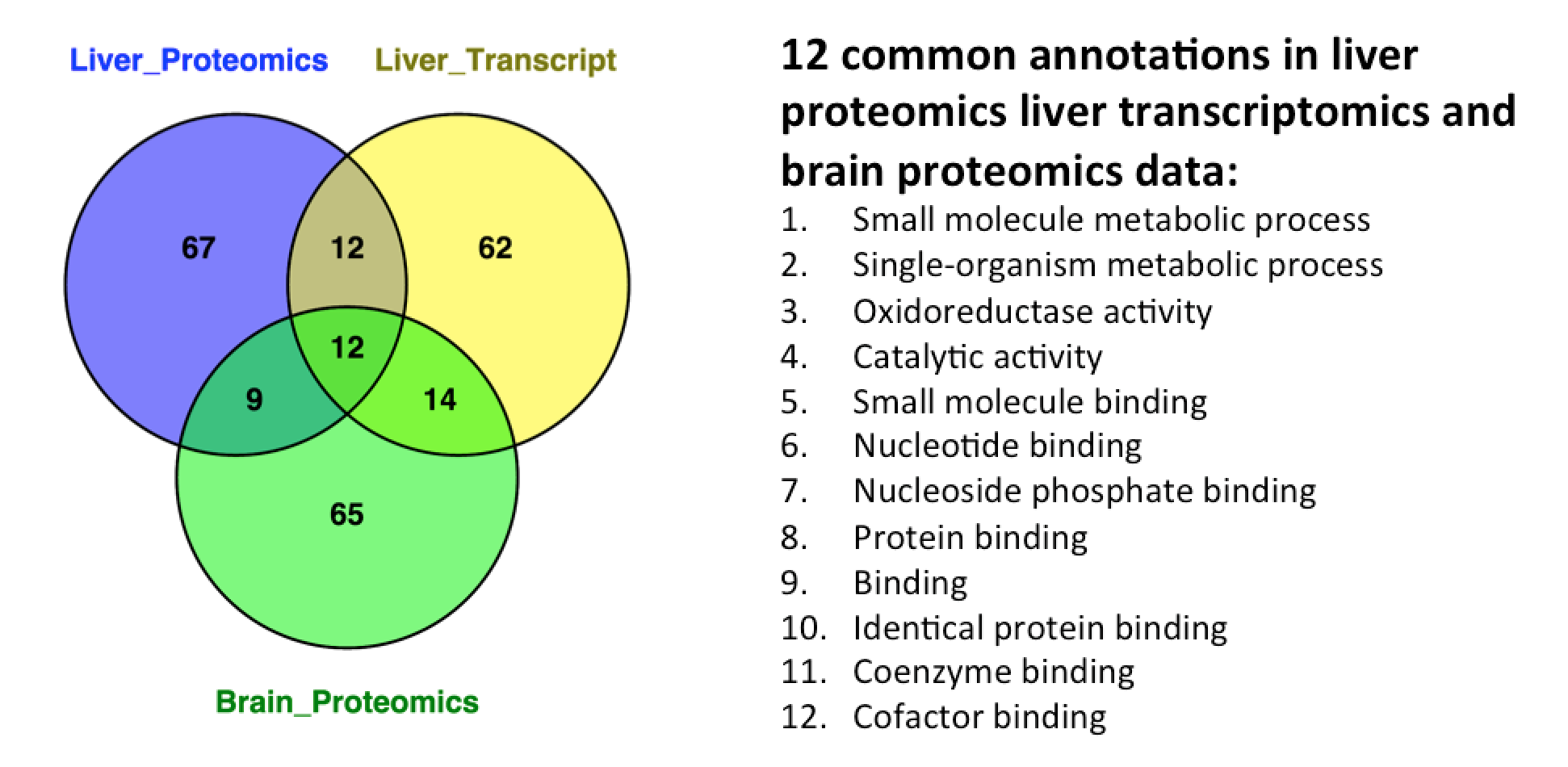


**Figure S7.** Venn diagram (left) showing shared annotations (right) among liver proteomics (125 proteins), liver trancriptomics (650 genes) and brain proteomics (40 proteins) dataset. Significantly enriched GO (BP, and MF) annotations (FDR < 0.05) in differentially regulated liver proteins (this study) and liver transcripts (Yadetie et al., 2013) and brain proteins (Berg et al., 2010) were compared, showing 12 common annotations.
